# Alcohol use and HIV serostatus of partner predict high-risk sexual behavior among patients receiving antiretroviral therapy in South Western Uganda

**DOI:** 10.1186/1471-2458-13-430

**Published:** 2013-05-03

**Authors:** Francis Bajunirwe, David R Bangsberg, Ajay K Sethi

**Affiliations:** 1 Department of Community Health, Mbarara University of Science and Technology, P.O. BOX 1410, Mbarara, Uganda; 2 Harvard Medical School, Boston, MA, USA; 3 Center for Global Health, Massachusetts General Hospital, Boston, MA, USA; 4 Department of Population Health Sciences, University of Wisconsin-Madison, School of Medicine and Public Health, Madison, WI, USA

**Keywords:** High-risk sexual behavior, Antiretroviral treatment, Rural and urban, Uganda

## Abstract

**Background:**

Antiretroviral treatment restores the physical and immunological function for patients with HIV/AIDS and the return of sexual desire. The frequency and correlates of sexual activity among patients receiving ART have not been widely studied. There is concern that widespread availability of ART may result in sexual disinhibition including practice of high-risk sexual behavior. We determined the correlates of sexual activity and high-risk sexual behavior in an ART-treated population in rural and urban Uganda.

**Methods:**

We conducted a cross-sectional study among 329 ART-treated adult patients at two hospitals, one located in rural and another in urban western Uganda. We collected data on sexual activity, frequency of condom use, pregnancy, viral load (VL) and CD4. Patients were considered sexually active if they had had sexual intercourse in the last 6 months. Any unprotected sex was considered high-risk sex. A two-stage logistic regression was performed to determine factors associated with sexual activity and high-risk sex among those sexually active.

**Results:**

Overall, 222 (67%) patients were women, 138 (41.2%) had been on ART for at least one year, and 168 (51.4%) were sexually active of whom 127 (75.6%) used condoms at the last intercourse. Younger age (<=30 years) (Odds ratio; OR=2.3, 95% CI 1.2, 4.2), higher monthly income (OR=4.1, 95% CI 2.4, 7.4), and being married (OR=22.7, 95% CI 8.2, 62.9) were associated with being sexually active. Undetectable VL, CD4 count and treatment duration were not significantly associated with sexual activity. Among the sexually active, alcohol consumption (OR=3.3, 95% CI 1.2, 9.1) and unknown serostatus of partner (OR=5.8, 95% CI 1.5, 21.4) were significant predictors of high-risk sexual behavior. The frequency of unprotected sex at the last intercourse was 25.9% and 22.1% among the men and women respectively and was not significantly different (*p* value for chi square test =0.59).

**Conclusion:**

Younger persons receiving ART are more likely to be sexually active. ART clients are more likely to engage in unprotected sex when sero-status of partner is unknown or report use of alcohol. Counseling on alcohol use and disclosure of sero-status may be useful in reducing high risk sexual behavior.

## Background

Antiretroviral therapy (ART) restores quality of life, physical functioning for majority of HIV-infected individuals [1-3] and return of sexual desire and resumption of sexual activity [4]. Safe sexual practice among HIV infected patients includes consistent use of condoms and is encouraged as part of positive living. There is widespread concern that large-scale availability of ART may result in sexual behavior disinhibition [5], possibly because HIV infected patients may perceive themselves as not infectious. In a U.S. study of patients attending primary HIV care, the belief that an undetectable viral load reduces infectiousness was significantly associated with high-risk behavior such as unprotected sexual intercourse [6]. Similarly, uninfected individuals may have lower perceived threat of HIV acquisition because of the availability of the treatments, believe that its use associated with lower infectiousness, and possibly engage in high-risk sexual behavior. Several studies have shown that initiation of ART does not necessarily result in risky sexual behavior, and in fact results in an increase in safer sexual practices [7], with up to 70% reduction in risky sexual behavior practices 6 months after initiation of ART. However the reports have been conflicting with some data suggesting that risky behavior may instead increase [5].

Based on the paradigm of treatment as prevention, one would expect that widespread use of ART will reduce the incidence of HIV transmission by reducing the community viral load [8]. However, mathematical models show effectiveness of combination prevention efforts will be dampened if large-scale treatment is accompanied with sexual behavioral disinhibition [9-11]. Population-based studies in Uganda show that there has been an increase in the number of casual partners accompanied by a decline in condom use among those in the middle age groups [12]. Data from Uganda also suggest that HIV incidence and prevalence are no longer on the decline as had previously been experienced [13], though the role of changing sexual behavior was not clearly explained.

The predictors of sexual activity and high-risk sexual behavior have not been adequately assessed among ART patients. This would be useful as it might identify individual-level risk factors to address during sexual behavior counseling. Specifically, alcohol consumption has been independently associated with HIV infection [14], adverse outcomes such as non adherence [15] and high-risk sexual behaviors in the general population [16], but fewer studies have been conducted among patients receiving ART particularly in resource-limited and rural settings. To determine these predictors, we conducted a cross-sectional study to determine the prevalence and correlates of sexual activity among patients receiving ART and to determine the factors associated with high-risk sexual behavior among those who are sexually active.

## Methods

### Study design and setting

We conducted a cross-sectional study among patients receiving ART at government supported health facilities in a rural (Sheema) and urban (Mbarara) district all in south western Uganda. Data were collected from government facilities in the district at Kitagata and Mbarara Hospitals located in Sheema and Mbarara districts, respectively. The two facilities are the largest hospitals in their respective districts; however, Sheema is rural and Mbarara is urbanized. Patients were eligible to participate if they had completed at least 6 months of antiretroviral therapy. Patients were enrolled consecutively and data was collected between April and October 2006.

Research assistants administered questionnaires to the study participants in the local language of Runyankore. Blood samples were also drawn at the same time to determine biological measurements. Data was then entered into Epidata and analyzed using STATA version 11 (StataCorp, College Station, Texas).

### Measurements and data analysis

We collected data on sexual activity, consistency of condom use, number of sexual partners and rating of current interest in sexual activity. Respondents were considered sexually active if they reported sexual intercourse in the last 6 months. Any unprotected sex was considered as high-risk sexual activity. To determine high-risk sexual behavior, participants were asked “The last time you had sexual intercourse, was a condom used?” Blood samples were collected to determine viral load and CD4 count. Viral load was considered undetectable if plasma HIV RNA concentration was <50 copies per milliliter (Roche, Amplicor). We compared baseline characteristics between the rural and urban sites using chi-square testing for the categorical variables and t-test for the continuous variables. We performed separate logistic regression analysis with sexual activity and unprotected sex as dependant variables and several factors including age, marital status, gender, place of residence (rural versus urban), monthly income, educational level, CD4 count, viral load, ART treatment duration and alcohol consumption were considered as predictors. We collected data on consumption of all alcohol containing beverages and any consumption of an alcohol containing beverage in the last 6 months was considered as an exposure. We report odds ratios and corresponding 95% confidence intervals.

### Ethics

The study was submitted to and approved by the Mbarara University of Science and Technology Institutional Review Board and final permission was obtained from the Uganda National Council of Science and Technology. We obtained informed written consent from all participants before they were enrolled in the study.

## Results

We interviewed a total of 329 patients at the two hospitals of the 334 that were approached. Of those who accepted to participate, 175 (53%) were at the rural site and the 154 (47%) were at the urban hospital (Table 1). Majority of the patients at both sites were female. Also, the two populations were similar in terms of literacy rates, monthly income and level of education. In comparison with patients at the rural site, those at the urban site had been on treatment for significantly longer, were less likely to drink alcohol and less likely to report a missed pill over the last three days, had higher proportion of patients with CD4 count above 200 cells per ml but a lower proportion of patients with undetectable viral load.

**Table 1:** Baseline characteristics of patients receiving antiretroviral treatment at Kitagata and Mbarara Hospitals in southwestern Uganda, April to October 2006

**Characteristic**	**Kitagata hospital**	**Mbarara hospital**	***p *value**
	**(Rural)**	**(Urban)**	
	**n=175**	**n=154**	
Aged =<30 years	28 (16.0)	28 (18.1)	0.25
Female	111 (63.4)	111 (72.1)	0.09
Can write	145 (82.8)	130 (83.4)	0.80
Can read	144 (82.3)	133 (85.1)	0.38
Marital status			
Widowed	74 (42.5)	54 (34.8)	
Separated/Divorce	21 (12.1)	17 (10.9)	
Never married	4 (2.3)	15 (9.7)	
Married Polygamous	18 (10.3)	12 (7.7)	
Married monogamous	57 (32.8)	57 (36.7)	0.04
Monthly income			
Less than 5 USD	85 (48.6)	63 (41.7)	
5 to 30 USD	49 (28.0)	45 (29.8)	
Over 30 USD	41 (23.4)	43 (28.5)	0.42
Education			
None	22 (12.6)	16 (10.5)	
Seven years or less	93 (53.1)	68 (44.4)	
More than 7 years	60 (34.3)	69 (45.1)	0.14
ART Treatment duration			
One year or less	134 (76.6)	58 (37.4)	
More than one year	41 (23.4)	97 (62.6)	0.001
Alcohol use in last 6 months	21 (12.0)	8 (5.2)	0.03
Non-Adherent using 3 day self report	26 (14.9)	5 (3.3)	<0.001
Viral load undetectable	147 (84.0)	108 (72.5)	0.012
CD4 cell count			
200 cells or less	48 (27.4)	19 (15.7)	
More than 200 cells	127 (72.6)	102 (84.3)	0.02

### Comparison of sexual behavior among males and females

Overall 168 (51.4%) patients were sexually active in the previous 6 months. Among the men, 75.7% of them were sexually active compared to 39.4% among the females and the difference was statistically significant (chi square p value <0.0001, see Table 2). The men were also more likely to report higher interest in sex and an increased interest in sexual activity since ART started.

**Table 2:** Comparison of sexual behavior characteristics among men and women receiving antiretroviral treatment in southwestern Uganda, April to October 2006

**Characteristic**	**Male**	**Female**	***p *value**
	**n=107**	**n=222**	
Sexually active	81 (75.7%)	87 (39.4%)	<0.0001
Current interest in sex			
Low	48 (44.9%)	155 (69.8%)	
Normal	56 (52.3%)	60 (27.0%)	
High	3 (2.8%)	7 (3.2%)	<0.0001
Interest in sex since ART			
started			
Decreased	36 (33.9%)	125 (56.3%)	
Remained the same	50 (47.2%)	77 (34.7%)	<0.0001
Increased	20 (18.9%)	20 (9.0%)	
	**Among sexually active only n=168**		
	**Male=81**	**Female=87**	
Mean number of lifetime sexual partners (median)	8.4 (3)	3.2 (2)	<0.001*
Mean number of sexual partners over the last 6 months (Median)	1.2 (1)	0.98 (1)	<0.001*
Unprotected sex at last intercourse (among sexually active only)	21 (25.9%)	19 (22.1%)	0.59
Sero status of partner at last			
intercourse	27 (33.3%)	5 (5.8%)	
Negative	37 (45.7%)	42 (48.8%)	
Positive	17 (21.0%)	39 (45.4%)	<0.001
Unknown			

* Wilcoxon-Mann–Whitney test.

Among the sexually active participants (n=168 or 51.4%), the men had a significantly larger number of lifetime sexual partners (8.4 versus 3.2 among men and women respectively, *p* value for Mann–Whitney test <0.001), and a larger number of sexual partners over the last six months (1.2 versus 0.8 among men and women respectively, *p* value for Mann–Whitney test <0.001). The frequency of unprotected sex at the last intercourse was 25.9% and 22.1% among the men and women respectively and was not significantly different (*p* value for chi square test =0.59). However, the women were more likely to report intercourse with a partner of unknown serostatus at their last intercourse compared to the men (45.4% versus 21.0% respectively, chi square p value <0.0001).

### Predictors of sexual activity and high-risk sexual behavior

The younger respondents (those aged 30 years or less) were more likely to engage in sexual activity compared to older patients. Women were 80% less likely to be sexually active compared to the men (OR=0.2, 95% CI 0.12, 0.35). In increasing order of magnitude, separated/divorced, never married, married polygamous and married monogamous were significantly more likely to be sexually active compared to the widowed (Table 3). Respondents with more education and in the higher income brackets were also more likely to be sexually active.

**Table 3:** Predictors of sexual activity and high risk sexual activity among patients receiving antiretroviral treatment at Kitagata and Mbarara Hospitals in southwestern Uganda, April to October 2006

	**Predictors of sexual activity among all participants n=330**		**Predictors of high risk sexual activity among sexually active n=168**	
**Characteristic**	**Odds ratio**	**(95% CI)**	**Odds ratio**	**95% CI**
Age				
Over 30 years	1.0		1	
30 years or younger	2.3*	1.2, 4.2	1.9	0.88, 4.3
Gender				
Male	1.0		1.0	
Female	0.2*	0.12, 0.35	0.81	0.39, 1.6
Marital status				
Widowed	1.0		1.0	
Separated/ Divorced	4.1*	1.8, 9.3	0.50	0.10, 2.4
Never married	6.3*	2.3, 17.6	0.93	0.17, 4.9
Married polygamous	22.7*	8.2, 62.9	0.89	0.24, 3.2
Married monogamous	40.6*	19.3, 85.2	0.60	0.20, 1.7
Residence				
Rural	1.0		1.0	
Urban	1.4	0.92, 2.3	0.95	0.46, 1.9
Years of Education				
None	1.0		1.0	
Less than 7 years	2.3*	1.1, 4.9	0.66	0.17, 2.5
More than 7 years	3.9*	1.8, 8.6	0.41	0.11, 1.5
Monthly income				
Less than 5 USD	1.0		1.0	
5 to 30 USD	2.4*	1.4, 4.1	0.64	0.26, 1.5
Over 30 USD	4.1*	2.4, 7.4	0.63	0.26, 1.5
CD4 count				
Less than 200	1.0		1.0	
200 and over	1.2	0.69, 2.1	1.3	0.50, 3.6
VL suppression				
Detectable	1.0		1.0	
Undetectable	0.41	0.12, 1.4	1.1	0.11, 11.6
3 day self report adherence				
Missed a pill	1.0		1.0	
Missed no pill	0.81	0.38, 1.7	0.58	0.18, 1.8
Duration on ART				
One year or less	1.0		1.0	
One to two years	1.36	0.82, 2.3	0.44	0.18, 1.1
More than 2 years	1.34	0.70, 2.6	0.59	0.20, 1.7
Alcohol use				
None in past 6 months	1.0		1.0	
Alcohol in past 6 months	1.9	0.85, 4.2	3.3*	1.2, 9.1
Serostatus of partner				
Negative			1.0	
Positive	-	-	2.5	0.66, 9.1
Unknown	-	-	5.8*	1.5, 21.4

*Significant at 0.05 level.

Among the sexually active respondents, alcohol use and serostatus of sexual partner at intercourse were significantly associated with high-risk sexual behavior. Any alcohol use in the past 6 months was associated with a three-fold increase in the odds of unprotected sex compared to no alcohol use (OR=3.3, 95% CI 1.2, 9.1). Respondents who had sexual partners of unknown serostatus had an almost 6-fold increase in the odds of unprotected sex compared to those partners who were known to be HIV negative (OR=5.8, 95% CI 1.5, 21.4). Overall, respondents with a partner of known serostatus (negative or positive) were less likely to report unprotected sexual intercourse at last sex compared to those with partners of unknown serostatus (p=0.004, chi square).

In a multivariable logistic regression model (Table 4) to predict high risk sexual behavior and adjusted for age and gender among the sexually active participants, alcohol use and serostatus of partner remained significant predictors.

**Table 4:** Multivariable logistic regression of factors associated with unprotected sex among sexually active respondents (n=168) receiving antiretroviral therapy at Kitagata and Mbarara Hospitals

**Predictor**	**Odds ratio**	**95% CI**	***p *value**
Any alcohol use in past 6 months (vs. no consumption)	3.7	1.3, 10.6	0.012
Serostatus of partner at last intercourse			
Negative	1.0	--	--
Positive	2.3	0.62, 9.1	0.21
Unknown	5.5	1.4, 21.5	0.01
Urban hospital (vs. rural)	0.93	0.43, 2.0	0.87
Age younger than 30	1.6	0.69, 3.7	0.27

## Discussion

Our study has shown that any alcohol consumption is associated with high risk sexual behavior among patients receiving antiretroviral therapy in a resource limited setting. These findings are in agreement with several other studies in the literature that have showed the association between alcohol use and high risk sexual behavior and have been combined in a recent meta-analysis [17]. However most of these studies were conducted in the US and other western countries and in fact only 4 of the 27 studies in the meta-analysis were African. Therefore, our study is one of the few studies that have demonstrated the association between alcohol use and high risk sexual behavior among ART clients in sub Saharan Africa. However, the association between alcohol and high-risk sexual behavior is well known and has been demonstrated in different sub groups such male sex workers in Mombasa [18], HIV+ women and men attending primary care clinics [19], fishing villages in Uganda [20,21] and hotel and bar workers in Tanzania [22] and among men who have sex with men (MSM) in Soweto, South Africa [23], few studies have assessed factors associated with high-risk sexual behavior or performed rural and urban comparisons in the same location.

This observational study in western Uganda has also shown that majority of patients receiving antiretroviral therapy are sexually active and sexual activity is especially higher among men than women. The high proportion of sexually active adults is consistent with other studies where the percentage was up to 70% [24]. The high frequency of sexual activity may be indicative of the restoration of physical functioning following ART which may be accompanied by the desire to live a normal life again which includes sexual activity[2]. However the sexual activity was accompanied by high-risk sexual behavior. The prevalence of unprotected sexual intercourse was over 20% among both men and women and very similar to a recent study in the urban informal settlement of Kibera in Nairobi, Kenya [25], baseline data in a rural Ugandan cohort [26] but slightly lower than a recent Ethiopian study [27] where more than one third of the ART clients reported risky sexual behavior.

However, our data indicate significant proportion of respondents reported decreased interest in sexual activity once they started ART. Majority of studies have shown a reduction in risky sexual behavior following initiation of ART [28], but to the best of our knowledge, none has shown decreased interest in sexual activity. Anecdotal evidence suggests this may be because a large number of these patients focus on their own recovery once they start on ART.

Our study showed no evidence of association between viral load suppression and high-risk sexual behavior. This finding is in agreement with several studies and the conclusions of a meta-analysis [29]. However, the meta-analysis comprised studies mostly from the developed countries. Since then, several studies among ART clients in sub Saharan Africa have also shown no evidence of the association between detectable or undetectable viral load and high-risk sexual behavior [30,31]. However, studies among MSM in the US have shown that perception of undetectable viral load [32], or reduced concern about HIV transmission following ART [33,34] are associated with higher frequency of unprotected sex. Such studies are yet to be conducted in sub Saharan Africa.

Alternatively, some studies have shown reduced odds of high-risk sexual behavior among patients who had viral load suppression or good adherence to ART [35]. The expectation is patients with viral load suppression will perceive themselves as non-infectious and therefore engage in high-risk sexual behavior.

Our data show that knowledge of sero-status of partner influenced condom use with respondents more likely to report unprotected sexual intercourse when sero-status of partner was not known. Studies elsewhere have shown contrasting findings with less risky sexual practice with partners of unknown sero-status [27]. Among South African couples, awareness of a partner’s HIV positive status and HIV positive concordance were associated with protected sex [36]. Yet some studies have shown no difference in risky sexual practice by sero-status of partner [37]. Disclosure of HIV sero-status to the main partner has been associated with safer sexual practices [38]. It should be noted that our study did not establish the nature of these partners, specifically casual versus regular partners and how these may influence use of condoms.

In a Kenyan [25] and Ugandan study [30], women were more likely to report inconsistent condom use compared to the men but this was not the case in our study. In fact there were no gender differences in the frequency of condom use. Also, unlike the study in Kenya, duration of time on ART did not predict the risk of unprotected sex. The reason for these differences is not clear but may be attributed to the nature of the populations under investigation. Majority of women in our study were leading more stable lives and may be less vulnerable compared to women in the informal settlements in the slums of Kibera in Kenya. However, women in our study were more likely to report intercourse with a partner of unknown sero status than men and this may be because of fear of disclosure and the power differential in sexual relationships in this setting. In an African traditional setting, women are unlikely to ask their husbands or partners to go for HIV testing but the opposite is true. This strengthens the cause for emphasizing positive prevention among ART clients and encouragement on disclosure of HIV test results to the partners.

Our study has several limitations, first is that we measured alcohol consumption as a dichotomous outcome. The data collection did not establish the quantities and grade of alcohol consumption so as to determine the dose response relationship. Additionally, our data were not able to support analysis to determine whether the reported high-risk sex was under the influence of alcohol. Second, the high-risk sexual behavior was self-reported and may have been underreported because of a social desirability bias. Third, our study is cross sectional and therefore limited with respect to causal inference. Also, we were unable to characterize the changes in sexual behavior within an individual as the respondents improve.

## Conclusion

In conclusion, our study has shown that younger persons receiving ART are more likely to be sexually active, ART clients are more likely to engage in unprotected sex when serostatus of partner is unknown and if they are consumers of alcohol. We recommend positive prevention and safe sex counseling particularly to clients who use alcohol and encourage disclosure of HIV serostatus among clients whose partners are of unknown status**.**

## Competing interests

The authors have no competing interests to declare.

## Authors’ contribution

FB conceived the idea, participated in data collection, analysis and drafting of the first version of this manuscript and revisions. AKS participated in conception of the idea, data analysis and reviewed the first draft of the manuscript. DRB contributed to conception of the idea, analysis and presentation of results and revision of the first draft of the manuscript. All authors reviewed and approved the final version of the manuscript.

## Pre-publication history

The pre-publication history for this paper can be accessed here:

http://www.biomedcentral.com/1471-2458/13/430/prepub
